# Twelve-Month Outcomes and Optical Coherence Tomography (OCT) Biomarkers After Intravitreal Dexamethasone Implantation in Pseudophakic Eyes with Post-Vitrectomy Cystoid Macular Edema (CME)—Refractory to Medical Therapy

**DOI:** 10.3390/diagnostics15020147

**Published:** 2025-01-10

**Authors:** Francesco Pignatelli, Alfredo Niro, Giuseppe Addabbo, Pasquale Viggiano, Giacomo Boscia, Maria Oliva Grassi, Francesco Boscia, Cristiana Iaculli, Giulia Maria Emilia Clima, Antonio Barone, Ermete Giancipoli

**Affiliations:** 1 Eye Clinic, Hospital “SS. Annunziata”, ASL Taranto, 74100 Taranto, Italy; pignatelli.oculista@gmail.com (F.P.); alfred.nir@tiscali.it (A.N.); addabbo.oculista@gmail.com (G.A.); 2 Department of Translational Biomedicine Neuroscience, University of Bari “Aldo Moro”, 70125 Bari, Italy; pasquale.viggiano90@gmail.com (P.V.); bosciagiacomo@gmail.com (G.B.); mariaolivagrassi@gmail.com (M.O.G.); francescoboscia@hotmail.com (F.B.); 3 Department of Ophthalmology, Policlinico Riuniti Foggia, University of Foggia, 71122 Foggia, Italy; cristiana.iaculli@unifg.it (C.I.); giuliaclimak@gmail.com (G.M.E.C.); antoniobarone79@yahoo.it (A.B.)

**Keywords:** post-surgical cystoid macular edema, small-gauge pars plana vitrectomy, intravitreal dexamethasone implant, OCT biomarkers, rhegmatogenous retinal detachment, epiretinal membrane

## Abstract

**Background**: In this study, we evaluated the incidence of cystoid macular edema (CME) after pars plana vitrectomy (PPV) for different retinal pathologies and assessed the role of optical coherence tomography (OCT) biomarkers in guiding treatment decisions in post-surgical CME patients who were refractory to medical therapy over a follow-up period of 12 months. **Methods**: Medical records of consecutive pseudophakic patients, who underwent PPV for different retinal pathologies, were retrospectively evaluated in this single-center, uncontrolled study. The incidence of post-PPV CME was assessed. Eyes with post-PPV CME in the first 2 months after surgery, with available clinical and OCT data for 12 months after surgery, were included in the evaluation. The mean best-corrected visual acuity (BCVA; logMAR), mean central macular thickness (CMT; μm) change, and response to different treatments [medical therapy and intravitreal dexamethasone (DEX) implant] were evaluated 1, 3, 6, 9, and 12 months after PPV. The impact of OCT biomarkers on the exposure to DEX implants was assessed. Adverse events, potentially related to the treatment, were investigated as well. **Results**: Of the 346 pseudophakic patients (352 eyes) who participated in this study, 54 (54 eyes) developed CME within the first 2 months after PPV (incidence of 15.3%). Among them, 48 patients were deemed eligible for the 12-month analysis. Preoperative mean BCVA (1.44 ± 0.99 logMAR) significantly improved to 0.32 ± 0.37 logMAR after 12 months (*p* < 0.001). The mean baseline CMT of 347 (±123.5) μm significantly decreased to 290 μm (±80.4; *p* = 0.003) by the end of the follow-up. Twenty-five eyes (52%) required one or more DEX implants for CME, due to being refractory to topical therapy. Significant correlations were found between the mean CMT values at various time points. Additionally, patients who required DEX implants at months 3 and 9 were more likely to present intraretinal fluid (IRF), disorganization of inner retinal layers (DRIL), disorganization of outer retinal layers (DROL), and hyper-reflective foci (HRF) at 1-month OCT. Five patients experienced a slight increase in intraocular pressure (IOP), which was successfully managed with topical medication. **Conclusions**: Topical therapy alone can be a valuable option for post-PPV CME in approximately 50% of patients. Significant visual recovery and macular thickness reduction at 12 months demonstrated that DEX implants can be a safe and effective second-line treatment for pseudophakic patients with post-PPV CME and who are refractory to medical therapy. Early post-surgical OCT biomarkers may indicate a more severe CME that might benefit from the steroid implant.

## 1. Introduction

Post-surgical cystoid macular edema (CME) is a pathological condition where inflammation-based damage to the intraretinal capillaries leads to the accumulation of serous fluid in the extracellular and intracellular spaces at the macula level [1,2]. It is considered the leading cause of reduced vision following both uneventful cataract and vitreoretinal surgery, with a reported incidence of 0.1–2.3% and between 10% and 47%, respectively [2,3,4].

This condition usually develops 4 to 12 weeks after surgery, with a peak postoperative incidence at 4–6 weeks. Although the precise pathogenetic mechanism is still poorly understood, subclinical inflammation, induced by surgical trauma, is deemed as the major player in CME development after surgery. The upregulation of inflammatory mediators, like prostaglandins, cytokines, and other vasopermeability factors, may lead to blood–retinal barrier breakdown with the consequent permeabilization of perifoveal capillaries and intraretinal fluid (IRF) accumulation [2,5].

Several conditions are recognized as potential risk factors for post-surgical CME onset. Systemic diseases, like diabetes and hypertension; intraoperative complications; and pre-existing ocular conditions, such as uveitis and diabetic retinopathy, seem to influence the development of the pathology [2,6,7].

This macular edema has a relatively benign natural history and spontaneously resolves within 6 months in more than 50% of eyes.

A prompt medical treatment, based on the use of topic medications, including steroidal and nonsteroidal anti-inflammatory drugs (NSAIDs), may be warranted in these patients to at least speed-up the resolution of retinal fluid build-up and to promote faster visual recovery [5]. Given the inflammatory nature of the condition, intravitreal steroids, including the intravitreal sustained-release dexamethasone (DEX) implant (Ozurdex^®^, Allergan Inc., Irvine, CA, USA, and Allergan Pharmaceuticals, Ireland), are extensively used for the treatment of post-surgical CME, especially in patients resistant to topical medications. Encouraging results emerged from several clinical studies that included patients treated with intravitreal DEX implants for CME, secondary to both cataract and vitreoretinal surgery [5,8,9,10,11].

Optical coherence tomography (OCT) is an essential diagnostic tool that enables the detection and accurate quantification of even subtle changes occurring at the macula in eyes with post-surgical CME. High-resolution OCT scans may provide key elements regarding the precise localization of the retinal fluid and the integrity of specific foveal structures such as the ellipsoid zone (EZ), external limiting membrane (ELM), and inner retinal layers. All these features are widely recognized as indicators of visual prognosis and responses to a given treatment in several macular pathologies, namely diabetic macular edema (DME), exudative age-related macular degeneration (AMD), and macular edema secondary to retinal vein occlusion (RVO) [12,13,14,15,16].

Although many studies focused on post-pars plana vitrectomy (PPV) CME, to the best of our knowledge, very little evidence can be found in the literature about the role of clinical characteristics and qualitative OCT features as potential biomarkers of response to intravitreal DEX implantation in pseudophakic eyes that developed a CME after small-gauge PPV [10].

The aim of the present study was to assess the incidence of CME in pseudophakic patients undergoing small-gauge PPV for different retinal pathologies. Twelve-month visual and anatomical outcomes, intravitreal DEX implant exposure and safety, and correlations between demographic and clinical characteristics, OCT structural biomarkers, and the need for DEX implant were evaluated as well.

## 2. Materials and Methods

In this retrospective, uncontrolled clinical study, medical and surgical records of pseudophakic patients undergoing small-gauge PPV for different retinal pathologies were reviewed. All surgeries were carried out at the “SS. Annunziata Hospital” Eye Clinic in Taranto, Italy, between January 2021 and July 2023. This study adhered to the tenets of the Declaration of Helsinki and was approved by the local Institutional Review Board (IRB).

To be included, patients had to fulfill the following inclusion criteria: age of 18 years or older; presence of a regularly signed informed consent for the surgery; pseudophakic status at the time of PPV with a documented minimum period between the previous cataract surgery and the vitreoretinal procedure of 1 year; uncomplicated small-gauge PPV for reasons of rhegmatogenous retinal detachment (RRD), epiretinal membrane (ERM), lamellar macular holes (LMHs), removal of silicone oil (SO), and vitreous hemorrhage (VH) secondary to a posterior vitreous detachment (PVD); a minimum follow-up of 12 months; a comprehensive description of the surgical procedure; clinical data and macular OCT scans (macular cube, radial, and raster scans with a line crossing the fovea center) available at 1, 3, 6, 9, and 12 months after surgery and fluorescein angiograms in the eyes that developed a central macular thickening during the postoperative period.

Patients with a history of exudative AMD, diabetic retinopathy, RVO, uveitis, and uncontrolled glaucoma were excluded. In cases of incomplete clinical data and poor-quality OCT scans that had too-low resolutions to allow a reliable qualitative assessment of the macular condition, patients were also excluded.

### 2.1. Surgical Procedure

All surgeries were performed by a single, experienced vitreoretinal surgeon (F.P.) on patients under peribulbar block (4 cc Ropivacaine + 1 cc Lidocaine) or general anesthesia. After a proper disinfection of the surgical field with 5% povidone-iodine (Oftasteril, Alfa Intes Industria Terapeutica Splendore S.r.l., Naples, Italy), a three-port 23- or 25-gauge (23-G; 25-G) PPV was carried out using the Constellation^®^ Vision System (Alcon Laboratories, Fort Worth, TX, USA). Silicone oil was aspirated from the vitreous chamber, then replaced with a balanced salt solution (BSS) or air, after multiple fluid–air exchanges to wash out any emulsion, in eyes scheduled for the removal of SO. The core vitrectomy and peripheral shaving were performed with a 7500-cut per min (cpm) cut rate and a linear aspiration of 0–650 mmHg in all other cases. Triamcinolone acetonide was systematically used for vitreous staining to ensure a better residual vitreous removal and to confirm posterior hyaloid detachment. Perfluorcarbon liquid (PFCL) was used to drain the subretinal fluid and stabilize the posterior retina only in RRD cases. In the presence of a proliferative vitreoretinopathy (PVR) complicating an RRD, the peeling of epiretinal and subretinal membranes after staining with Trypan Blue (TB) 0.15% + Brilliant Blue G (BBG) 0.05% + Lutein 2%solution (DOUBLE DYNE; Alfa Intes Industria Terapeutica Splendore S.r.l., Naples, Italy), was attempted with vitreoretinal microforceps. Relaxing retinotomies and retinectomies were usually reserved to cases of RRD with severe PVR. Fluid–air exchange was performed to flatten the retina immediately after all the vitreous tractions were removed. Endolaser retinopexy was applied to all the retinal breaks and along the borders of the retinotomies and retinectomies. Then, 24% sulfur hexafluoride (SF6), 14% octafluoropropane (C3F8), and SO (1000 centistokes, 5000 centistokes or heavy SO) were alternatively used as the endotamponade, according to the complexity of the cases.

After core vitrectomy, peripheral shaving, and PVD induction, the ERM and internal limiting membrane (ILM) were removed with end-grip vitreoretinal forceps, after staining with TB 0.15% + BBG 0.05% + Lutein 2% solution, in patients with idiopathic ERM and LMHs. After ERM + ILM removal, the peripheral retinal was carefully evaluated under scleral depression in all cases to rule out the presence of peripheral retinal breaks and micro-holes requiring endolaser retinopexy. A partial fluid–air exchange was then carried out. Sclerotomies were systematically closed with 8.0 Vicryl sutures to reduce the risk of early postoperative hypotony secondary to leaking wounds.

### 2.2. Definition and Diagnosis of Post-Surgical Macular Edema

**Figure 1:**
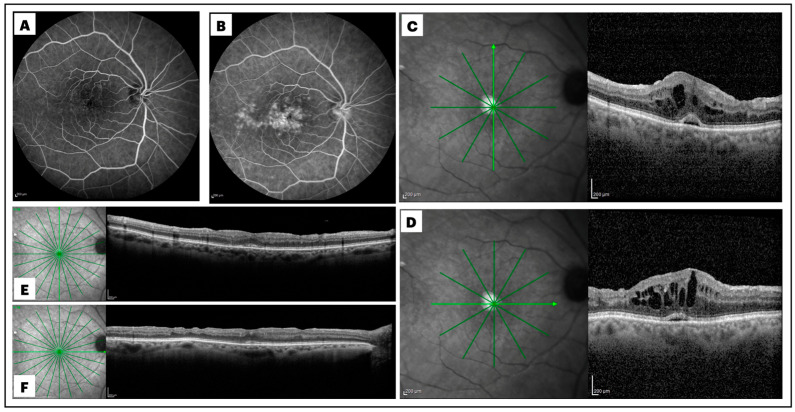
Early (**A**) and late (**B**) frames of a fluorescein angiogram, along with macular spectral domain optical coherence tomography (SD-OCT) scans (**C**,**D**), of a patient who developed cystoid macular edema (CME) one month after small-gauge pars plana vitrectomy (PPV) for rhegmatogenous retinal detachment repair. Extensive macular leakage was observed during the late phase of the angiogram, with a diffuse and irregular macular hyperfluorescence (**B**). Late-phase optic disk hyperfluorescence was also evident (**B**). The vertical (**C**) and horizontal (**D**) SD-OCT B-scans crossing the foveal center showed significant central macular thickening, the presence of intraretinal hyporeflective cysts, and a subfoveal hyporeflective cuff of fluid. Subsequent vertical (**E**) and horizontal (**F**) SD-OCT B-scans demonstrated a significant reduction in central macular thickness, with complete resolution of intraretinal and subretinal fluid, 3 months after intravitreal dexamethasone administration.

Post-PPV CME was defined by an increase in macular thickness of ≥10% of the previous OCT value and/or by the presence of more than three circular or ovoid intraretinal hyporeflective spaces in the inner and/or outer layers in the central millimeter of the Early Treatment Diabetic Retinopathy Study (ETDRS) grid and by the identification of subretinal fluid (SRF) accumulation at the foveal location [4]. The diagnosis of post-surgical macular edema needed to be further supported by the evidence of late-phase macular and optic disc hyperfluorescence at postoperative fluoresceine angiography (Spectralis HRA  +  OCT; Heidelberg Engineering, Heidelberg, Germany) (Figure 1).

### 2.3. Analysis of the Biomarkers

**Figure 2:**
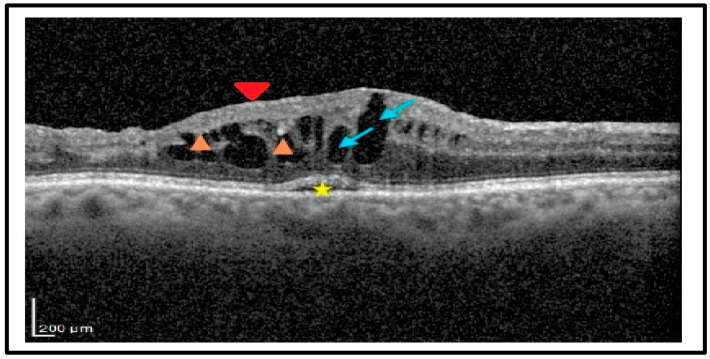
Spectral domain optical coherence tomography (SD-OCT) at month 1, revealed a macular edema with large intraretinal cysts (blue arrows), subretinal fluid at the fovea (star), hyper-reflective foci (HRF, orange arrowheads), and disorganization of inner retinal layers, (DRIL, red arrowhead).

Qualitative OCT assessment, carried out by two independent expert observers (A.N. and E.G.) on OCT scans taken one month after surgery, led to the recognition of the following structural biomarkers: intraretinal cysts (IRCs), defined by the presence of circular or ovoid intraretinal hyporeflective spaces in the inner and/or outer layers; SRF, indicating a subfoveal hyporeflective “cuff” of fluid beneath the neuroepithelium; disorganization of inner retinal layers (DRIL), defined by the complete loss of boundaries between the ganglion cell, inner plexiform, and outer nuclear plexiform layers at the foveal location; disorganization of outer retinal layers (DROL), featured by the complete loss of the EZ + ELM hyper-reflective bands at the subfoveal location; hyper-reflective foci (HRF), identified as dot-like lesions, less than 30 μm in size, with the same reflectivity of the retinal nerve fiber layer (Figure 2).

The main outcomes evaluated were the incidence of CME in pseudophakic eyes after small-gauge PPV, the change in the baseline mean best-corrected visual acuity (BCVA) and central macular thickness (CMT) at 12 months in the subset of patients who developed a form of post-vitrectomy CME and were consequently treated, and the safety of intravitreal DEX.

Secondary outcomes were the correlations between the exposure to intravitreal DEX implant during the follow-up and specific demographic, clinical, and OCT characteristics.

### 2.4. Treatment of Post-Surgical Macular Edema

Medical therapy, consisting of a combination of topical steroids (preservative-free DEX eye drops 1 mg/mL, administered 4 times daily) plus topical NSAIDs (Bromfenac eye drops 0.9 mg/mL, twice daily), was used for at least one month, in all patients who developed post-surgical CME.

**Figure 3:**
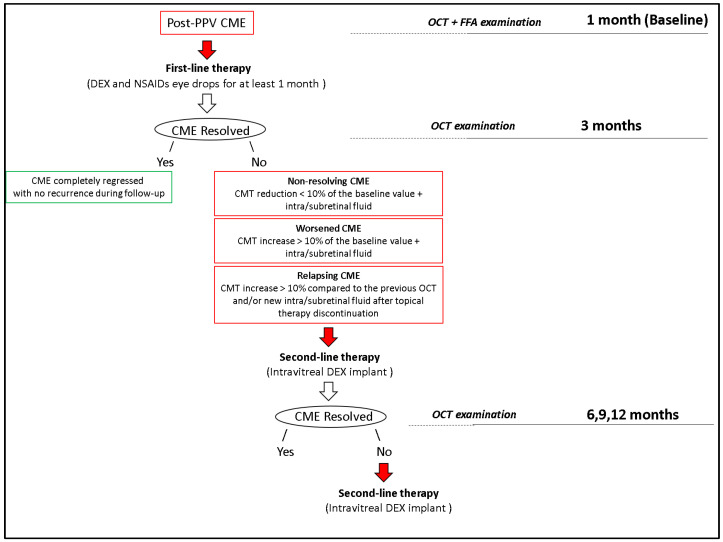
Observation and therapy flow chart. PPV, pars plana vitrectomy; CME, cystoid macular edema; OCT, optical coherence tomography; FFA, fundus fluorescein angiography; DEX, dexamethasone; NSAID, nonsteroidal anti-inflammatory drug; CMT, central macular thickness.

A DEX implant of 0.7 mg, injected into the vitreous cavity starting from month 3, according to the standard protocol [17], was reserved to all cases that did not respond to the medical treatment or where CME tended to relapse after topical treatment discontinuation (Figure 3).

### 2.5. Statistical Analysis

The qualitative variables are presented as frequencies and percentages, while the quantitative data are presented as means ± standard deviations. No formal sample size calculation was performed. For assessing the change in BCVA and CMT over the follow-up period, the non-parametric test known as the Wilcoxon rank-sum test was used. For frequencies and percentages, the Fisher test was used. All statistical tests were performed at the *p* < 0.05 significance level. Univariate and multivariate regression models were performed to assess the relationship between DEX implants at 3, 6, and 9 months and each independent variable. The independent variables included gender, age, retinal disease, CMT, and OCT biomarkers. The independent variables that were significant in the univariate regression analysis were considered in multiple regression models. Statistical analysis was made using STATA 12.1 Statistical Software (StataCorp), 2014, release 12 (College Station, TX, USA).

## 3. Results

Medical records of 346 consecutive pseudophakic patients (352 eyes), who underwent small-gauge PPV for RRD (235 eyes), SO removal (43 eyes), ERM (62 eyes), or non-diabetic VH (12 eyes), between January 2021 and July 2023, were identified. Gradable OCT scans, taken between the first and the second month after surgery, were available for all of them. Post-PPV CME, defined as a central foveal thickening with evidence of IRF and/or SRF, in the absence of any epiretinal component, and further confirmed by the late-phase macular and optic disc hyperfluorescence at fluoresceine angiography, developed in 54 patients (incidence: 15.3%) within the first two months after surgery.

Of these 54 patients (54 eyes), 6 were excluded for the following reasons: the follow-up was shorter than 6 months in 4 eyes, and some relevant clinical data were missing in the remaining 2 eyes.

Ultimately, 48 eyes (21 right/27 left) of 48 consecutive patients (15 women and 33 men; mean age: 64.1 ± 4.3 years; range: 57–73) were deemed eligible for the final analysis. Indication for PPV in this population were RRD repair in 36 eyes (75%), 1000 centistokes SO removal in 10 eyes (20.8%), and ERM peeling in 2 eyes (4.1%). Considering only the subgroup of patients who underwent vitreoretinal surgery for RRD repair, the macula was detached in 31 eyes (86.1%), PVR was evident in 14 eyes (38.8%), and a mild VH, that did not impair a proper OCT evaluation of the macula during the preoperative period, was present in the remaining 2 eyes (5.5%).

The mean time (±SD; range) between the previous cataract surgery and the vitreoretinal procedure was 3.8 ± 2.3 years (range 1–11) in the study population. A comprehensive clinical assessment, together with reliable OCT scans, were available at 1, 3, 6, 9, and 12 months for all of them.

Additional relevant demographic and baseline characteristics are listed in Table 1.

**Figure 4:**
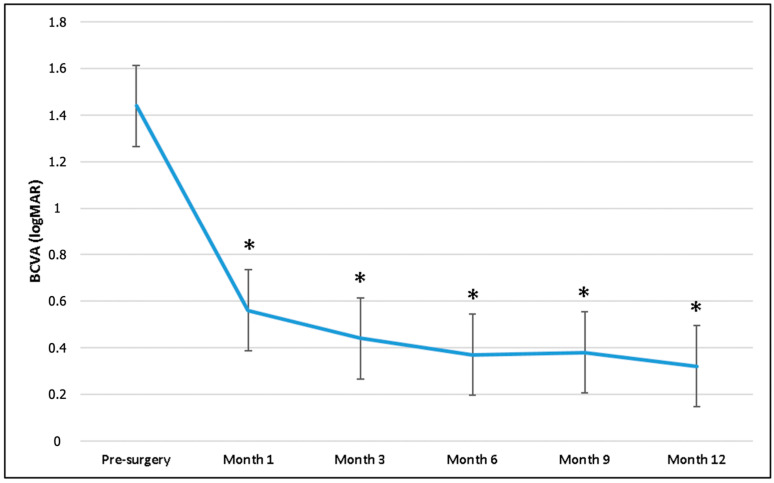
Best-corrected visual acuity (BCVA) changes during follow-up. * *p*-value, Wilcoxon test (BCVA baseline as reference value).

Preoperative mean BCVA (1.44 ± 0.99 logMAR) significantly improved at 1 (0.56 ± 0.40 logMAR), 3 (0.44 ± 0.48 logMAR), 6 (0.37 ± 0.35 logMAR), 9 (0.38 ± 0.39 logMAR), and 12 months (0.32 ± 0.37 logMAR) in the overall population (*p* < 0.001 for all time points) (Figure 4).

**Figure 5:**
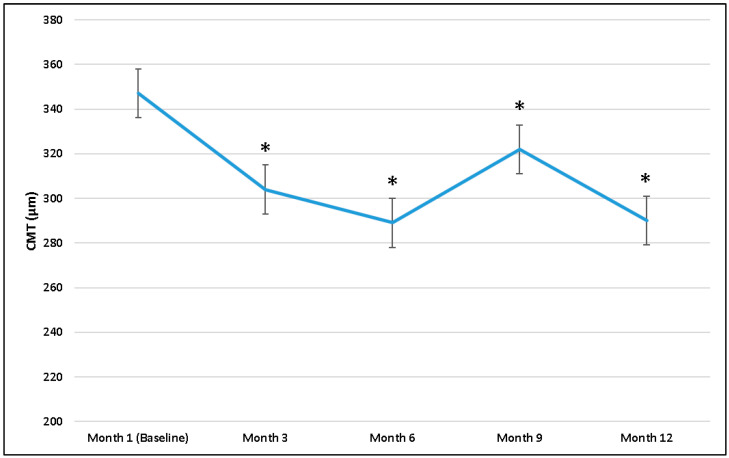
Central macular thickness (CMT) changes during follow-up, starting from 1 month. * *p*-value, Wilcoxon test (CMT 1 month as reference value).

The mean CMT was 347 ± 123.5 μm at 1 month after PPV, and it significantly decreased to 304 ± 93.6 μm at month 3, 289 ± 79.7 μm at month 6, 322 ± 104.5 μm at month 9, and 290 ± 80.4 μm at the last visit. Mean CMT changes at all time points are reported in Figure 5.

The qualitative analysis of OCT at month one disclosed the presence of IRF in 34 eyes and SRF in 14 eyes. Additionally, the disorganization of inner and outer retinal layers (EZ + ELM) at the foveal location and the presence of HRF were evident in 11 (23%), 10 (20.8%), and 9 (18.7%) eyes, respectively. The complete analysis of the OCT biomarkers in month one is reported in Table 2.

### 3.1. Treatment Exposure

All patients were treated with topical therapy (DEX eye drops 4 times daily + NSAIDs eye drops 3 times/daily for one month) as first-line treatment. Intravitreal DEX implants could be administered starting from month 3, only in cases of non-resolving (CMT reduction < 10% of the baseline value with persistence of IRF or SRF), worsened (CMT increase > 10% of the baseline value with the presence of IRF or SRF), or relapsing (CMT increase > 10% when compared to the OCT of the previous evaluation and/or evidence of new SRF and IRF) CME.

CME completely regressed after topical medications in 23 eyes (47.9%), without any evidence of recurrence during the entire course of the follow-up. The remaining 25 eyes (52%) required one or more intravitreal DEX implants at some point during the follow-up due to the persistence or recurrence of macular edema.

DEX implant was administered at 3, 6, 9, and 12 months in 16 (33.3%), 5 (10.4%), 16 (33.3%), and 4 (8.3%) eyes, respectively. Each patient treated with DEX implants received a mean number of 1.64 ± 0.56 (range, 1–3) implants between months 3 and 12. More specifically, 10 patients (40%) received a single implant, 14 patients (56%) received two implants, and 1 patient (4%) was treated with three implants (Table 3).

The mean interval between two consecutive injections was 5.2 ± 1.37 (range, 3–6 months) in patients who required more than one administration.

**Figure 6:**
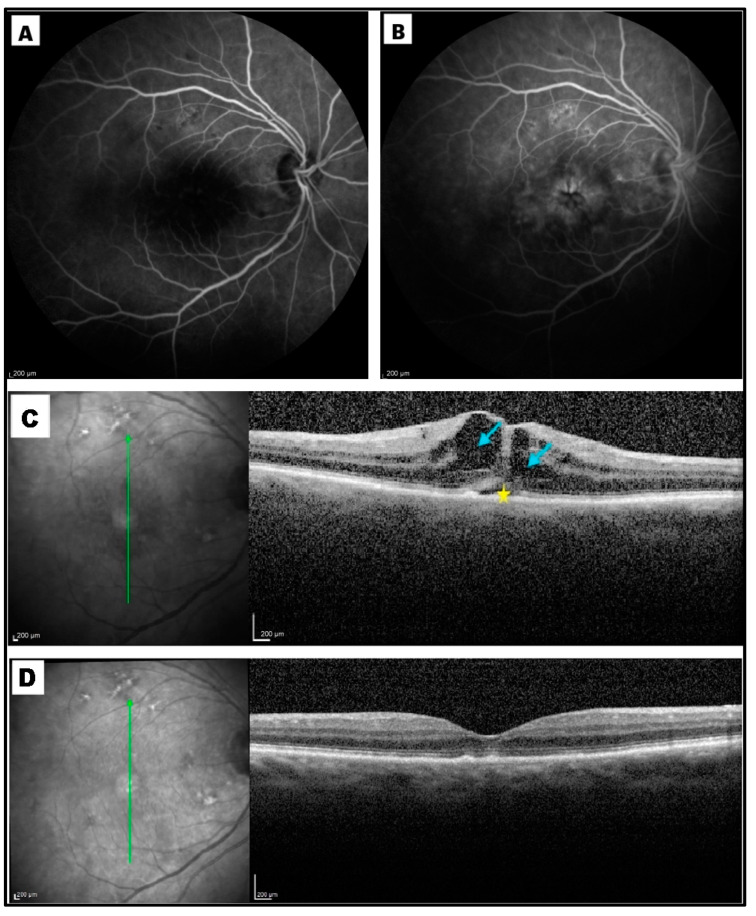
Post-vitrectomy cystoid macular edema (CME) after surgery for rhegmatogenous retinal detachment in the right eye of a 65-year-old lady. (**A**) Early- and (**B**) late-phase fluorescein angiography, performed one month after surgery, showed the classical perifoveal petaloid staining pattern and late leakage of the optic disc. (**C**) Optical coherence tomography (OCT) scan taken at month 1 revealed the presence of intraretinal cysts (blue arrows) and subretinal fluid (yellow star). (**D**) At month 3, OCT revealed a complete resolution of intraretinal and subretinal fluid after topical therapy. Best-corrected visual acuity improved from 20/200 to 20/30; central macular thickness decreased from 366 µm to 205 μm.

Two representative cases were reported in Figure 1 and Figure 6.

### 3.2. Analysis of the Correlations

The correlation analysis, between the mean CMT values taken at different time points, revealed the existence of a statistically significant correlation between mean CMT at month 1 and month 3 (*p* < 0.001). Additionally, both the mean CMTs at month 3 and 6 significantly correlated with the mean CMTs at months 9 and 12 (*p* < 0.001), while the mean CMT at month 9 significantly correlated with the mean CMT at the last visit (month 12; *p* < 0.001) (Table 4).

A univariate analysis using a logistic regression model was performed to look at the potential correlations between the need for DEX implants at month 3 and specific demographic and clinical characteristics and predefined structural OCT biomarkers. The indication to start treatment with the steroid implant at month 3 significantly correlated with the mean CMT (*p* = 0.005) and the presence of IRC (*p* = 0.04), DRIL (*p* = 0.004), and HRF (*p* = 0.02) in the 1-month OCT scans.

When the same univariate logistic regression model was tested with treatment (DEX implant) at month 6, no statistically significant correlations could be found. None of the tested variables appeared to be significantly correlated with the exposure to the implant at month 3 in the multivariate logistic regression model.

When the probability of receiving an implant at month 9 was tested for different clinical and OCT variables, a statistically significant correlation with HRF and DROL emerged both in the univariate (HRF: OR = 5.80, *p* = 0.02; DROL: OR = 7.51, *p* = 0.01) and multivariate (HRF: OR = 5.53, *p* = 0.04; DROL: OR = 7.11, *p* = 0.03) logistic regression models. The results of univariate and multivariate analysis are reported in Table 5.

### 3.3. Safety

No severe ocular and non-ocular adverse events were reported for patients who underwent one or more intravitreal DEX administrations. Baseline mean (±SD) intraocular pressure (IOP; 15.6 ± 3.9 mmHg) at month one did not change significantly during the entire course of the follow-up (Table 6).

A total of 5 patients (20%) presented with mild IOP elevation (>25 mmHg) that was successfully managed with topical IOP-lowering medications at months 6 (3 patients) and 12 (2 patients). No patients developed glaucoma or required filtering surgery for a chronic, refractory IOP elevation.

## 4. Discussion

Limited information is available about the incidence of CME after vitrectomy or phacovitrectomy. Leisser et al. analyzed data from six studies and showed that the incidence of new IRF, detected 3 months after surgery, was 6% and 3% after phacovitrectomy and vitrectomy alone, respectively [18,19].

Post-PPV CME occurred in 15.3% of our pseudophakic patients, in line with the data reported in the literature ranging from 3.8% to 17.1%, according to the underlining pathology that led to surgery or the surgical procedure itself (vitrectomy vs phacovitrectomy) [4,19,20,21].

Cataract surgery is a well-known risk factor for Irvine–Gass syndrome due to surgical manipulation in the anterior segment, which may promote the upregulation of inflammatory mediators, leading to a breakdown of the blood–retinal barrier and increased vascular permeability [19], and thus a potential source of bias when assessing the true incidence of post-PPV CME [20]. So, all patients included in the present study were pseudophakic at the time of vitrectomy, with a minimum time between the previous cataract surgery and the vitreoretinal procedure, as short as one year. However, excluding phakic eyes scheduled for a phacovitrectomy procedure may have reduced the chance of overrating the true incidence of post-surgical CME. 

The exact mechanism behind CME after vitrectomy is not well understood. Studies suggested several risk factors, including age over 50, multiple surgeries, severe PVR, aphakia, the type of endotamponade used, and the extent of endolaser treatment or cryopexy during PPV for RRD repair [22,23,24].

Intraretinal microcystoid changes were observed after PPV combined with macular peeling in patients with idiopathic ERMs, especially in those cases with pre-existing IRC [20], stage 4 ERMs, and candidates for a phacovitrectomy procedure [3]. It was proposed that the degree of retinal architectural disruption, found in end-stage ERMs, may make the retinal tissue more prone to the mechanical stress induced by ILM peeling maneuvers. This may cause a Muller cell disfunction, secondary to a retrograde trans-synaptic degeneration of inner retinal layers and an inflammatory process, with consequent impairments in fluid resorption at the macula. According to this theory, the CME observed after ILM peeling for ERMs may derive more from glial degeneration rather than a true blood–retinal barrier disfunction [3,25,26].

The reported incidence of CME after SO endotamponade ranges between 13.6% and 36%, depending on several factors such as SO viscosity, the grade of PVR complicating the retinal detachment, and the interval between PPV and SO removal. Both inflammatory and mechanical components were postulated as predisposing factors for CME formation in SO-filled eyes where SO can promote an inflammatory reaction by impairing the potassium ion buffering function of Muller cells. Furthermore, the adhesive interaction between the SO bubble and the retinal surface may translate into tractional forces to the macula, which may promote IRF accumulation. If spontaneous CME resolution was observed after SO removal in most eyes, some patients experienced further macular thickening after SO removal. This indicates that the pro-inflammatory stimulus may persist for some time, even after the elimination of SO [22].

Taken all together, these data suggest that, regardless of the initial retinal condition treated with PPV, many risk factors can contribute to increased intraocular inflammation [4].

Patients who underwent PPV for RRD repair (36) represented most of our study population (75%), followed by patients scheduled for SO removal (10 eyes, 20.8%) and idiopathic ERM peeling (2 eyes, 4.1%). Considering the relatively low number of patients included, especially in the ERM peeling group, and the heterogeneity of the retinal pathologies, we did not look at potential risk factors for post-PPV CME. Additionally, the present study was designed to assess the medical management of post-PPV CME not responding to first-line topical therapy, rather than to identify risk factors for this condition.

A low-grade inflammatory response can be considered the leading event that promotes retinal fluid accumulation in eyes that develop a form of post-PPV CME. Inflammatory mediators released during surgery may easily spread in the vitreous chamber, causing a blood–retinal barrier breakdown and extravasation of fluid from retinal vessels, as confirmed by the late leakage and staining of the optic nerve head and the macula, observed at the fluorescein angiography in these patients [2,3]. Furthermore, retinal glial dysfunction, induced by surgical trauma and again by inflammation, may concurrently promote the collection of fluid in the macula of these patients [3,25,27].

The paramount role of inflammation can be inferred by the satisfactory response to a given anti-inflammatory therapy in these patients [22].

Medical treatment, based on the administration of steroid and NSAID eyedrops, is generally considered a safe and effective option in patients with post-surgical CME. The good ocular penetration, combined with a low rate of adverse events, make topical therapy a fairly good first-line option in these cases [5]. Patients who failed to respond to medical treatment, and cases where CME tended to relapse as soon as the topical medication discontinued may benefit from more invasive treatments like intravitreal steroids [28,29] or anti-vascular endothelial growth factor (VEGF) agents [30].

Intravitreal DEX implant is a biodegradable corticosteroid implant, which provides a sustained release of 700 μg DEX into the vitreous for up to 6 months [9]. The results from different clinical studies showed that DEX implants can promote a significative long-term CMT reduction and visual acuity improvement in vitrectomized eyes that develop CME after RRD surgery and ERM peeling, both in naïve and refractory to medical therapy eyes [8,9,11,31].

Twenty-five eyes (52%) did not show a satisfactory response to the medical therapy in our study, so they were candidates for DEX implantation starting from month 3. BCVA improvement and significant CMT reduction indicated that intravitreal therapy was effective in promoting macular fluid resolution and visual recovery in this population.

While CME resolution was achieved after a single injection in 10 patients (40%), the remaining 15 patients required two or more injections by the end of the 12-month follow-up, with a mean number of 1.64 treatment per patient and a mean interval between two consecutive injections of 5.2 months. The maximum mean CMT reduction was reported at month 6, three months after the injection, with a recurrence of macular edema at month 9 in 10 patients (62.5% of the population that received the first injection at month 3).

This observation seems in line with similar reports in the literature [10,32]. The maximum effect of DEX implants is usually observed after one month of administration, with a progressive loss in effect in the following months. This can explain why approximately 44% of patients need additional implants after a mean period of 6 months [9,32].

The correlation of OCT structural biomarkers with visual function and their role as a predictor of the response to medical treatment in several macular pathologies has grown in popularity over the past few years. The existence of potential correlations between mean CMT values, measured at different time points, and between the DEX implant exposure and specific demographic, clinical characteristics, and predefined structural OCT biomarkers, were investigated in our study.

The mean CMT at month 1 significantly correlated with the mean CMT at month 3, while CMT values at months 3 and 6 both correlated with the mean CMTs at months 9 and 12. These correlations suggest most of the patients who displayed a more pronounced macular thickening at first month, still have a clinically significant CME at month 3, despite the medical therapy. Additionally, the correlations between the mean CMT at months 3 and 6 with that measured at months 9 and 12 may suggest that CME tends to relapse approximately 6 months after implantation. A greater CMT at month 1 suggests a more severe CME that may not resolve completely after medical therapy. Patients who presented with a thicker macula 1 month after vitrectomy continued to display persistent fluid build-up at month 3, thus requiring intravitreal treatment.

Very little evidence on the role of clinical and structural biomarkers as indicators of response to DEX implant, in patients with post-PPV CME, can be found in the literature.

Fressinger et al. investigated the efficacy of DEX implants in eyes that developed CME after PPV for ERM or macula on RRD and tried to identify functional and morphological OCT predictors of response. Their results indicated that the presence of SRF, the integrity of EZ, and a worse baseline BCVA were all predictive of a better functional outcome after implantation [10]. Furthermore, the presence of SRF and HRF were shown to be predictive of a better anatomical and functional response to DEX implants in DME [33,34,35].

The univariate logistic regression model revealed that the indication for DEX implant at month 3 significantly correlated with the mean CMT at month 1 and the existence of IRF, DRIL, and HRF at 1-month OCT. The need for a second implant at month 9 was significantly correlated with the presence of HRF and DROL at the OCT, both in the univariate and multivariate linear regression models. The presence of HRF and DROL at the OCT indicates an 8- and 6-fold increase, respectively, of the chance to be treated with an additional DEX implant, 9 months after surgery, for the recurrence of CME.

HRFs, considered the aggregation of activated microglia cells, located in both the inner and outer retina of patients with macular edema secondary to different retinal etiologies [33], are known to be associated with increased inflammation in the retina [35,36,37].

Conflicting evidence on the role of HRF as biomarkers of response to intravitreal steroids for DME still exists. Some authors demonstrated that the presence of HRF is associated with an excellent response to intravitreal steroids [35], while other reports showed that baseline numbers of HRF may be a predictive indicator of the early recurrence of macular edema after intravitreal DEX implantation for DME [38].

Accepting the above-mentioned evidence, a higher baseline number of HRF may indicate a more severe retinal inflammation with a more pronounced macular edema that may be resistant to topical therapy. This hypothesis could explain why patients who presented with a higher number of HRF at baseline did require intravitreal implant more frequently in our study.

It is worth noting that our assumption is purely speculative since no precise evidence of the impact of HRF on the response intravitreal steroid treatment in patients with post-PPV CME can be found in the literature to date.

The roles that IRF, DRILS, and DROL may play in defining the response to intravitreal steroids in these patients are less clear, with no conclusive evidence that can be derived from the literature.

We hypothesized that the presence of IRC, DRIL, and DROL at baseline may be suggestive of a more severe CME, with a higher degree of intraretinal degeneration. In a recent report, the odds of having DRIL was greater in DME eyes with an increased retinal thickness at the fovea [39]. The presence of large cystoid spaces in the inner retinal layers may also contribute to generating distortion in the retina, thus opening the way to DRIL. Conversely, the absence of DRIL was significantly correlated with the presence of SRF, which is known to be more commonly seen in acute DME [40]. This might indicate DRIL as a sign of chronic macular edema. Taken together, these observations may support the hypothesis that eyes with post-PPV CME, associated with the presence of IRF, DRIL, and DROL at the OCT, may be affected by a more severe condition that requires a more aggressive treatment rather than topical therapy alone.

When a multivariate logistic regression model was carried out, none of the tested variables correlated with the exposure to the intravitreal implant at month 3. The disagreement between the two models could be explained, at least in part, by the relatively reduced number of cases included in our study.

Repeated treatments with DEX implants over a prolonged period may generate some safety concerns, especially pertaining to the risk of uncontrolled IOP elevation. IOP rise > 25 mm Hg was reported in approximatively 26% of patients who underwent implantation for different retinal conditions. Overall, more than 90% of eyes with IOP elevation can be successfully managed with medical therapy, with only 0.5% eyes required filtering surgery [41].

An IOP increase > 25 mmHg was evident in 20% of the patients in our study, and it was medically managed in all cases. No cases of severe ocular adverse events like endophthalmitis, retinal detachment, or vitreous hemorrhage were reported.

The relatively small sample size, particularly patients affected by ERM, the retrospective nature, the lack of a control group and the heterogeneity of the retinal pathologies that required surgery, represent the major limitations of the present study. Additionally, correlations between clinical OCT biomarkers and visual function were not investigated. This is mainly due to the fact we feel that at least some degree of inner and outer retinal disruption (DRIL and DROL) observed in some patients may be related to the pre-existing retinal pathology rather than to post-vitrectomy CME.

To the best of our knowledge, this is the first study where the role of specific OCT biomarkers, such as DRIL, IRF, DROL, and HRF, as predictors of exposure to intravitreal DEX implants in pseudophakic eyes that developed CME after a vitreoretinal procedure, was investigated.

The strict inclusion criteria (in which only pseudophakic patients could be enrolled), the long follow-up, the availability of comprehensive clinical and OCT data for all the enrolled patients, and the use of fluorescein angiography to confirm the diagnosis of post-surgical CME further increase the reliability of our results.

## 5. Conclusions

In conclusion, approximatively 50% of pseudophakic patients, who developed CME after vitreoretinal surgery, may benefit from topical therapy alone. Intravitreal DEX implantation seems to warrant a sustained macular thickness reduction and visual recovery in patients who show an incomplete response to topical medications after 2 months of the diagnosis. The identification of specific biomarkers at the OCT performed one month after surgery may indicate a more severe CME and could help the clinician to stratify patients who will probably require an intravitreal therapy in the future.

## Figures and Tables

**Table 1:** Demographic and baseline characteristics.

		RRD	SO	ERM	*p*-Value *
*N*° (%); patients/eyes		36 (75)	10 (20.8)	2 (4.2)	
Laterality: right/left		16/20	6/4	1/1	0.29
Age, year (mean ± SD)		64.6 ± 4.5	63.8 ± 3.6	58.5 ± 0.7	0.17
Gender, *N*° (%)					
	Male	25 (69.4)	6 (60)	1 (50)	
	Female	11 (30.6)	4 (40)	1 (50)	
Type 2 Diabetes (controlled with therapy); *N*° (%)		3 (8%)	1 (10%)	0 (0%)	0.45
Time between cataract surgery and PPV; years, mean ± SD (range)		4.1 ± 2.48 (1–11)	3 ± 1.5 (2–6)	2 ± 0.7 (1–2.5)	0.20
Macula attached at the time of surgery (RRD group); *N*° (%)		31 (86.1%)	-	-	
PVR present: *N*° (%)		14 (38.8%)	-	-	
Mild VH associated with RRD; *N*° (%)		2 (5.5%)	-	-	
Endotamponade agent used (RRD group)					
	Gas (SF6 24%; C3F8 14%)	22 (64.7%)			
	Silicone Oil 1000 cst	10 (29.4%)			
	Densiron	2 (5.8%)			

RRD: rhegmatogenous retinal detachment; SO: silicone oil; ERM: epiretinal membrane; PVR: proliferative vitreoretinopathy; SF6: sulfur hexafluoride; C3F8: octafluoropropane; cst: centistokes. %, percentage calculated on the overall study population; * *p*-value, Fisher test.

**Table 2:** Optical coherence tomography (OCT) biomarkers.

	IRC	DRIL	DROL	HRF	SRF
RRD *n* (%)	20 (41.7)	7 (14.6)	8 (16.7)	7 (14.6)	7 (14.6)
SO *n* (%)	10 (20.8)	4 (8.3)	2 (4.2)	2 (4.2)	4 (8.3)
ERM *n* (%)	2 (4.2)	0 (0.0)	0 (0.0)	0 (0.0)	1 (2.1)
*p*	0.02	0.29	0.75	0.78	0.30

IRC, intraretinal cysts; DRIL, disorganization of inner retinal layers; DROL, disorganization of outer retinal layers; HRF, hyper-reflective foci; SRF, subretinal fluid; RRD, rhegmatogenous retinal detachment; SO, silicone oil; ERM, epiretinal membrane. %, percentage calculated on the overall study population. *p*-value, Fisher test.

**Table 3:** Intravitreal dexamethasone (DEX) implant performed at each time point.

	RRD	SO	ERM	*p*
DEX-i at 3 months, *n* (%)	10 (20.8)	5 (10.4)	1 (2.1)	0.37
DEX-i at 6 months, *n* (%)	3 (6.4)	2 (4.3)	0 (0.0)	0.52
DEX-i at 9 months, *n* (%)	11 (22.9)	5 (10.4)	0 (0.0)	0.31
DEX-i at 12 months, *n* (%)	4 (8.3)	0 (0.0)	0 (0.0)	0.49

RRD, rhegmatogenous retinal detachment; SO, silicone oil; ERM, epiretinal membrane; %, percentage calculated on the overall study population; *p*-value, Fisher test.

**Table 4:** Correlation between mean central macular thickness (CMT) at different time points.

		CMT 1 Month	CMT 3 Months	CMT 6 Months	CMT 9 Months	CMT 12 Months
CMT 1 month	Spearman’s Rho	—				
	*p*	—				
CMT 3 Months	Spearman’s Rho	0.592 ***	—			
	*p*	<0.001	—			
CMT 6 Months	Spearman’s Rho	0.174	0.274	—		
	*p*	0.237	0.060	—		
CMT 9 Months	Spearman’s Rho	0.145	0.541 ***	0.578 ***	—	
	*p*	0.326	<0.001	<0.001	—	
CMT 12 Months	Spearman’s Rho	0.117	0.573 ***	0.454 **	0.662 ***	—
	*p*	0.428	<0.001	0.001	<0.001	—

** *p* < 0.01, *** *p* < 0.001, Spearman’s Rank test.

**Table 5:** Univariate (A) and multiple (B) logistic regression model of intravitreal dexamethasone implant (DEX-i) at different time points on demographic and clinical variables.

**(A) DEX-i at 3 months as dependent variable**				
**Parameters**	**β**	**SE (β)**	***p***	**OR**
Age	0.10	0.07	0.17	1.11
Gender (male)	−3.67 × 10^−16^	0.66	0.99	0.99
Disease				
SO—RRD	0.95	0.73	0.19	2.6
ERM—RRD	0.95	1.46	0.51	2.6
CMT 1 month	0.01	0.003	0.005	1.01
IRC (No)	1.69	0.83	0.04	5.44
DRIL (No)	2.27	0.78	0.004	9.66
HRF (No)	1.76	0.79	0.02	5.80
DROL (No)	1.44	0.74	0.05	4.20
SRF (No)	0.95	0.68	0.16	2.60
** (B) ** ** DEX-i at 3 months as dependent variable **				
** Parameters **	** β **	** SE (β) **	*** p ***	** OR **
Age	0.09	0.1 0	0.35	1.1 0
Gender (male)	−0.18	0.87	0.83	0.83
Disease				
SO—RRD	0.49	0.98	0.61	1.64
ERM—RRD	0.77	2.44	0.75	2.17
CMT 1 month	0.008	0.005	0.09	1 .0
IRC (No)	−0.33	1.26	0.78	0.71
DRIL (No)	1.62	0.92	0.08	5.08
HRF (No)	1.33	1.03	0.19	3.8 0
** (A) DEX-i at 6 months as dependent variable **				
** Parameters **	** β **	** SE (β) **	*** p ***	** OR **
Age	0.04	0.1 0	0.69	1.03
Gender (male)	0.39	0.97	0.68	1.5
Disease				
SO—RRD	0.98	0.99	0.32	2.66
ERM—RRD	−15.2	2797.4	0.99	2.51 × 10^−7^
IRC (No)	−0.28	0.96	0.76	0.75
DRIL (No)	−17.7	3242.4	0.99	2.04 × 10^−8^
HRF (No)	1.23	1 .00	0.21	3.42
DROL (No)	0.06	1.18	0.96	1.06
SRF (No)	−0.22	1.17	0.84	0.8 0
**(A) DEX-i at 9 months as dependent variable**				
** Parameters **	** β **	** SE (β) **	*** p ***	** OR **
Age	0.02	0.07	0.74	1.02
Gender (male)	0.42	0.64	0.51	1.5
Disease				
SO—RRD	0.87	0.73	0.23	2.27
ERM—RRD	−15.7	1696.7	0.99	1.45 × 10^−7^
CMT 3 months	0.006	0.003	0.07	1 .00
CMT 6 months	0.007	0.004	0.11	1 .00
IRC (No)	0.14	0.65	0.82	1.15
DRIL (No)	0.67	0.7 0	0.33	1.97
HRF (No)	1.76	0.79	0.02	5.8 0
DROL (No)	2.02	0.78	0.01	7.51
SRF (No)	0.95	0.68	0.16	2.6 0
**(B) DEX-i at 9 months as dependent variable**				
** Parameters **	** β **	** SE (β) **	*** p ***	** OR **
Age	−0.02	0.09	0.85	0.97
Gender (male)	0.01	0.83	0.99	1.01
Disease				
SO—RRD	1.01	0.84	0.22	2.75
ERM—RRD	−14.9	1696.7	0.99	3.23 × 10^−7^
HRF (No)	1.71	0.9 0	0.04	5.53
DROL (No)	1.96	0.87	0.02	7.11
** (A) DEX-i at 12 months as dependent variable **				
** Parameters **	** β **	** SE (β) **	*** p ***	** OR **
Age	−0.20	0.15	0.18	0.81
Gender (male)	−0.33	1.19	0.77	0.71
Disease				
SO—RRD	−17.5	3400.7	0.99	2.39 × 10^−8^
ERM—RRD	−17.5	7604.2	0.99	2.39 × 10^−8^
CMT 3 months	−0.01	0.01	0.21	0.98
CMT 6 months	0.007	0.004	0.1 0	1 .00
CMT 9 months	0.005	0.004	0.2 0	1 .00
IRC (No)	−1.97	1.2 0	0.1 0	0.14
DRIL (No)	−17.4	3242.4	0.99	2.62 × 10^−8^
HRF (No)	−17.4	3584.6	0.99	2.78 × 10^−8^
DROL (No)	0.26	1.21	0.83	1.29
SRF (No)	−17.4	3104.4	0.99	2.54 × 10^−8^

SO, silicone oil; RRD, rhegmatogenous retinal detachment; ERM, epiretinal membrane; CMT, central macular thickness; IRC, intraretinal cysts; DRIL, disorganization of the inner retinal layers; DROL, disrupted retinal outer layers; HRF, hyper-reflective foci; SRF, subretinal fluid; SE, standard error; OR, odds ratio.

**Table 6:** Intraocular pressure (IOP) changes over follow-up.

Intraocular Pressure (IOP, mmHg) at Different Time Points	mmHg	*p*
IOP Baseline *	15.6 ± 3.9	
IOP 1 Month	15.6 ± 2.8	0.90 ^†^
IOP 3 Months	15.0 ± 2.4	0.32
IOP 6 Months	15.9 ± 3.1	0.31
IOP 9 Months	15.8 ± 2.1	0.71
IOP 12 Months	15.6 ± 1.9	0.80

IOP: intraocular pressure. * mean ± SD; ^†^ *p*-value, Wilcoxon test (IOP baseline as reference value).

## Data Availability

All raw data will be made available upon request.

## List of Abbreviations

AMD	age-related macular degeneration
BBG	brilliant blue G
BCVA	best-corrected visual acuity
BSS	balanced salt solution
CME	cystoid macular edema
CMT	central macular thickness
C3F8	octafluoropropane
DEX	dexamethasone
DME	diabetic macular edema
DRIL	disorganization of inner retinal layers
DROL	disorganization of outer retinal layers
ELM	external limiting membrane
ERM	epiretinal membrane
ETDRS	Early Treatment Diabetic Retinopathy Study
EZ	ellipsoid zone
HRF	hyper-reflective foci
ILM	internal limiting membrane
IOP	intraocular pressure
IRB	Institutional Review Board
IRC	intraretinal cysts
IRF	intraretinal fluid
LMHs	lamellar macular holes
LogMAR	Logarithm of the Minimum Angle of Resolution
NSAIDs	nonsteroidal anti-inflammatory drugs
OCT	optical coherence tomography
PFCL	perfluorcarbon liquid
PVD	posterior vitreous detachment
PPV	pars plana vitrectomy
RVO	retinal vein occlusion
PVR	proliferative vitreoretinopathy
RRD	rhegmatogenous retinal detachment
SD-OCT	spectral domain OCT
SF6	sulfur hexafluoride
SO	silicone oil
SRF	subretinal fluid
TB	trypan blue
VEGF	vascular endothelial growth factor
VH	vitreous hemorrhage
